# Vital Signs: Health Burden and Medical Costs of Nonfatal Injuries to Motor Vehicle Occupants — United States, 2012

**Published:** 2014-10-10

**Authors:** Gwen Bergen, Cora Peterson, David Ederer, Curtis Florence, Tadesse Haileyesus, Marcie-jo Kresnow, Likang Xu

**Affiliations:** 1 Division of Unintentional Injury Prevention, National Center for Injury Prevention and Control, CDC; 2 Division of Analysis, Research, and Practice Integration, National Center for Injury Prevention and Control, CDC; 3 McNeal Professional Services

## Abstract

**Background:**

Motor vehicle crashes are a leading cause of death and injury in the United States. The purpose of this study was to describe the current health burden and medical and work loss costs of nonfatal crash injuries among vehicle occupants in the United States.

**Methods:**

CDC analyzed data on emergency department (ED) visits resulting from nonfatal crash injuries among vehicle occupants in 2012 using the National Electronic Injury Surveillance System – All Injury Program (NEISS-AIP) and the Healthcare Cost and Utilization Project National Inpatient Sample (HCUP-NIS). The number and rate of all ED visits for the treatment of crash injuries that resulted in the patient being released and the number and rate of hospitalizations for the treatment of crash injuries were estimated, as were the associated number of hospital days and lifetime medical and work loss costs.

**Results:**

In 2012, an estimated 2,519,471 ED visits resulted from nonfatal crash injuries, with an estimated lifetime medical cost of $18.4 billion (2012 U.S. dollars). Approximately 7.5% of these visits resulted in hospitalizations that required an estimated 1,057,465 hospital days in 2012.

**Conclusions:**

Nonfatal crash injuries occur frequently and result in substantial costs to individuals, employers, and society. For each motor vehicle crash death in 2012, eight persons were hospitalized, and 100 were treated and released from the ED.

**Implications for Public Health:**

Public health practices and laws, such as primary seat belt laws, child passenger restraint laws, ignition interlocks to prevent alcohol impaired driving, sobriety checkpoints, and graduated driver licensing systems have demonstrated effectiveness for reducing motor vehicle crashes and injuries. They might also substantially reduce associated ED visits, hospitalizations, and medical costs.

## Introduction

Motor vehicle crashes are a leading cause of injury and death. Previous research has shown that motor vehicle crashes result in substantial mortality, with 22,912 motor vehicle occupants killed in 2012 in the United States (1), and an estimated 265,000 years of potential life lost in 2011 (CDC’s Web-Based Injury Statistics Query and Reporting System [WISQARS], unpublished data, 2014). The estimated medical cost of such fatalities was $226 million (2). Because the burden of nonfatal injuries caused by motor vehicle crashes has been less well-documented, this report estimates the U.S. health burden and medical and work loss costs of nonfatal motor vehicle crash injuries; the most recent available data on emergency department (ED) visits and hospitalizations were examined.

## Methods

Data from the 2012 National Electronic Injury Surveillance System – All Injury Program (NEISS-AIP), which is operated by the U.S. Consumer Product Safety Commission in collaboration with CDC, and data from the 2012 Healthcare Cost and Utilization Project National Inpatient Sample (HCUP-NIS) of the U.S. Agency for Healthcare Research and Quality were analyzed. NEISS-AIP is a nationally representative stratified probability sample of 63 U.S. hospitals (3). Detailed data on initial ED visits per injury per person are abstracted from medical records for all nonfatal injuries and poisonings. Patients who made more than one ED visit because of a crash injury in 2012 were counted separately for each visit. NEISS-AIP data are publicly available through CDC’s WISQARS (2). HCUP-NIS is based on a 20% stratified sample of inpatient hospital discharges at U.S. community hospitals. In 2012, 44 states participated in HCUP-NIS, and resulting data were weighted to provide national estimates (4). Data on work-related crash injuries were obtained from the NEISS-Work occupational supplement, which uses the same sample as NEISS-AIP. In all data sources, nonfatal occupant (driver or passenger) injuries from unintentional motor vehicle traffic crashes (hereafter called crash injuries) were defined consistent with the *International Classification of Diseases, Ninth Revision, Clinical Modification* external cause-of-injury codes E810–E819 with suffixes “.0” and “.1” (indicating injuries specific to motor vehicle occupants). Nature of injury categories (e.g., sprains/strains and fractures) were derived from the NEISS-AIP principal diagnosis codes. Rates of ED visits were calculated for all crash injuries using population estimates from the U.S. Bureau of the Census ( http://www.census.gov/population/projections/data/national/2012.html ), and for work-related crash injuries using estimates of full-time-equivalent (FTE) employees from the U.S. Bureau of Labor Statistics’ Current Population Survey ( http://www.census.gov/cps/methodology ).

Estimated counts, rates per 100,000 population, and 95% confidence intervals (CIs) for total, treated and released, and transferred or hospitalized (hereafter referred to as hospitalized) ED patients and the proportion of hospitalized ED patients were stratified by sex and age group. The age groups, selected to coincide with distinct crash risk and opportunities for intervention, were: 0–14 years, 15–29 years (further divided into 15–17 years, 18–20 years, 21–24 years, and 25–29 years), 30–39 years, 40–49 years, 50–59 years, 60–69 years, 70–79 years, and ≥80 years. Crude injury rates were presented for each age group, whereas overall and sex-specific injury rates were age-adjusted to the standard year 2000 population (2). For work-related crash injuries, the age group of 20–69 years was used to coincide with the ages of those most likely to drive for work. Differences in estimates were considered statistically significant (p≤0.05) if their CIs did not overlap. The proportion of ED visits by nature of injury were calculated using 2010 data (the most recent data available ). The annual estimated total number of hospital days was calculated by multiplying the total number of ED visits resulting in hospitalization from NEISS-AIP by the average length of stay from HCUP-NIS.

Methods for estimating lifetime medical and loss of work costs associated with crash injuries are described in detail elsewhere (5). The medical estimates included the cost of initial ED visits and hospitalizations for crash injuries, attributable lifetime medical costs (e.g., follow-up ED visits and hospitalizations, ambulance transportation, ambulatory care, prescription drugs, home health care, vision aids, dental visits, and medical devices), and nursing home and insurance claims administration costs. The loss of work estimates included lost expected employment earnings, lost fringe benefits, and lost value of household work. Costs beyond the first year after the crash injury were discounted at the recommended 3% (6). Medical costs were estimated from 2010 U.S. dollars (USD) data and inflated to 2012 USD using the Price Indexes for Personal Consumption Expenditures by Function from the U.S. Bureau of Economic Analysis (5). Work loss estimates are presented as 2012 USD based on the Employment Cost Index, Total Compensation, Civilian from the U.S. Bureau of Labor Statistics for productivity loss (5). Total lifetime medical costs were calculated by multiplying the number of treated and released ED patients or hospitalized patients by the corresponding average estimated lifetime medical cost for both sexes and each age group and summing the results.

## Results

During 2012, an estimated 2,519,471 ED visits (CI = 2,041,225–2,997,717) for crash injuries occurred, corresponding to an estimated rate of 806 visits per 100,000 population (Table 1). Of these visits, 1%–2% were identified as work-related, with a rate of 25 visits per 100,000 FTE employees. Age-specific rates by disposition did not vary significantly by sex. Total visit rates varied significantly by age; children aged 0–14 years had the lowest rate (281 visits per 100,000 population [CI = 218–344]), teens and young adults aged 15–29 years the highest rate (1,448 visits per 100,000 population [CI = 1,165–1,742]), and adults aged 30–39 years the second highest rate (1,075 visits per 100,000 population [CI = 883–1,267]) (Table 1). Rates for work-related crashes did not vary significantly by age group, ranging from 23 to 29 visits per 100,000 FTE employees aged 20–69 years.

**FIGURE 1:**
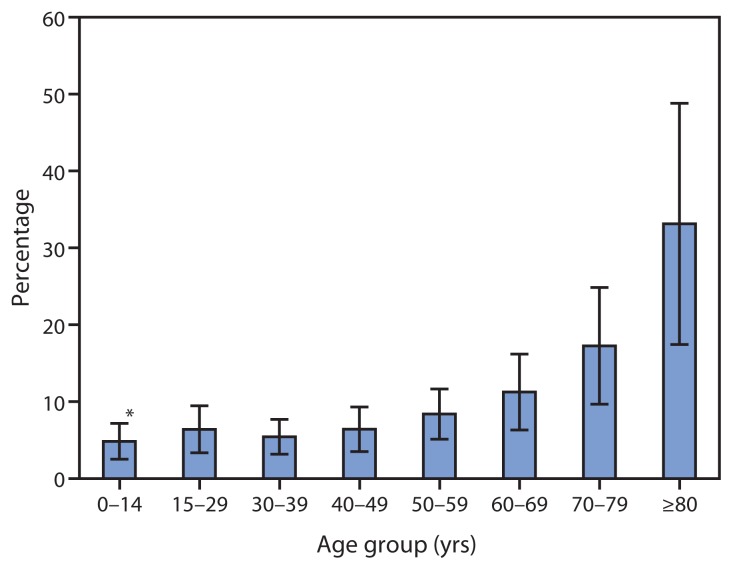
Percentage of emergency department visits for nonfatal crash injuries among motor vehicle occupants that result in hospitalization, by age group — National Electronic Injury Surveillance System, United States, 2012 * 95% confidence interval.

**FIGURE 2:**
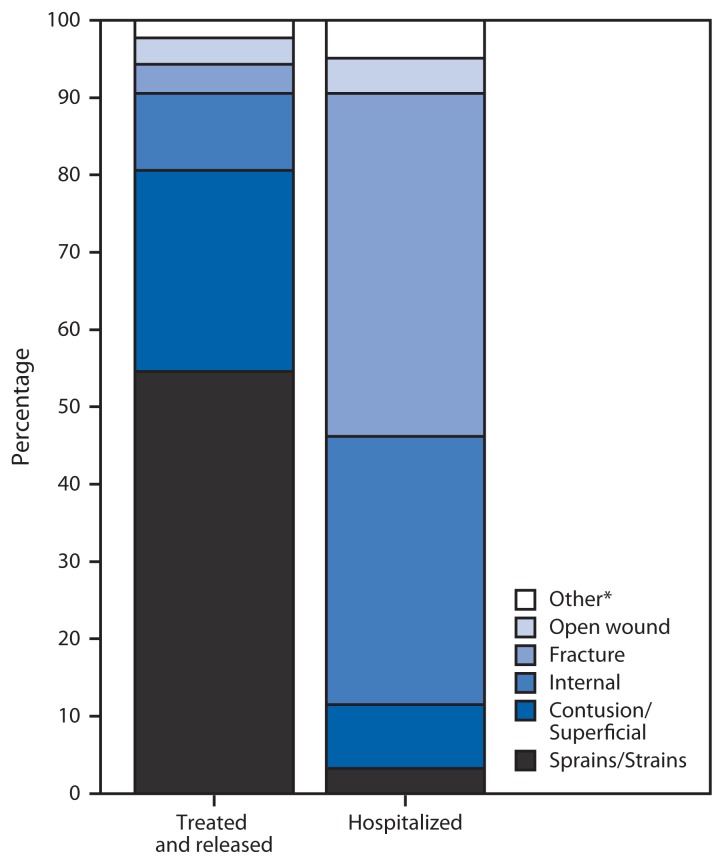
Percentage of emergency department visits for the top five nonfatal crash injuries among motor vehicle occupants, by nature of injury and disposition — National Electronic Injury Surveillance System, United States, 2010 * Estimates based on ≤20 injury cases or a national (weighted) estimate of ≤1,200 cases might be unstable.

Approximately 7.5% (N = 188,833 [CI = 110,377–267,288]) of persons visiting EDs because of crash injuries were hospitalized. A similar proportion of persons with work-related crash injuries (8%) were hospitalized. Adults aged ≥80 years had a significantly higher hospitalization rate (33%) than other age groups except for adults aged 70–79 years (17%) (Figure 1). The average length of stay for hospitalization among all ages was 5.6 days for a total of 1,057,465 hospital days. Sprains/ strains accounted for 55% of treated and released ED visits (Figure 2), although such injuries were the least likely to result in hospitalization, with 99.6% of patients with sprains/strains treated and released. Fractures accounted for just 4% of treated and released ED visits but resulted in hospitalization in 45% of cases.

The lifetime medical cost of crash injuries was estimated to be $18.4 billion: $7.7 billion for treated and released patients and $10.7 billion for hospitalized patients (Table 2). The average lifetime medical cost per hospitalized patient was $56,674 (Table 2). The average lifetime medical cost per treated and released patient was $3,362 (Table 2). The lifetime cost of work loss because of crash injuries in 2012 was estimated to be $32.9 billion: $9.4 billion for treated and released patients, and $23.5 billion for hospitalized patients. Crash injuries declined in the past decade. Compared with 2002, an estimated 397,761 fewer ED visits and 5,771 fewer hospitalizations occurred in 2012. This reduction was associated with an averted $1.7 billion lifetime medical cost and $2.3 billion work loss costs.

## Conclusions and Comment

The health burden and medical costs resulting from nonfatal crash injuries in the United States are substantial. In 2012, an estimated 2.5 million ED visits occurred because of such injuries, of which approximately 188,000 were serious enough to require hospitalization. This is equivalent to 6,902 ED visits and 517 hospitalizations every day. With U.S. households averaging 5.7 vehicle trips per day, the risk for these injuries is widespread (7).

Motor vehicle crashes result in substantial mortality and years of potential life lost. This study shows that the nonfatal injury burden is also high. For each motor vehicle occupant killed in a crash in 2012, eight were hospitalized, and 100 were treated and released from the ED. The estimated lifetime medical cost of nonfatal crash injuries is similar to other serious, but perhaps more well-known, public health problems. For example, the estimated lifetime medical cost of crash injuries is approximately 50% higher than the estimated $12.6 billion cost for human immunodeficiency virus (HIV) in the United States (8). On average, each crash-related ED visit costs $3,362, and each hospitalization costs $56,674. These nonfatal crash injury costs can create both an immediate and lifelong burden for individuals and their families, as well as employers, and public and private health care payers. Although these are lifetime medical costs, the majority of medical costs (approximately 75%–90%) are estimated to occur in the first 18 months after the crash (5). In addition to the burden of medical costs, crash injuries cause a substantial lost lifetime productivity valued at $32.9 billion.

Teens and young adults aged 15–29 years accounted for a disproportionate share of the burden, comprising 21% of the population but accounting for 38% of both the treated and released visits and costs in this analysis. Other studies have shown that this age group has a higher prevalence of risk factors for crash injuries. In 2012, teens and young adults aged 16–24 years had the lowest prevalence of observed restraint use (80%) compared with all other age groups (87%–88%) (9). In 2010, adults aged 21–24 years and 25–34 years had the highest self-reported prevalence of driving after having had too much to drink (3.6% and 2.6%, respectively) compared with adults aged 18–20 years (2.2%) and adults aged ≥35 years (0.8%–1.9%) (10).

Older adults in this study were more likely to be hospitalized for a crash injury compared with other age groups. Increased frailty, rather than increased risk for crash involvement, likely accounts for the majority of increased fatality risks for adults aged ≥60 years (11), and might explain the increased proportion of ED visits that result in hospitalization among this age group.

Analyses of risk factors such as nonuse of restraints, alcohol use, and geographic location were not possible in this study. Although the Fatality Analysis Reporting System (derived from police reports) has national and state-level information on motor vehicle crash fatalities, including factors contributing to the crash, no single data source exists for risk factors and associated medical outcomes for nonfatal crash injuries. Also, the completeness of external cause-of-injury coding in existing state-based hospital discharge and ED data systems varies, making it difficult to monitor and assess motor vehicle crash injuries treated in hospitals in some state and local jurisdictions (12,13).

The findings in this report are subject to at least four limitations. First, NEISS-AIP and HCUP-NIS use different data collection methods and thus report different estimates of the number of crash injuries. NEISS-AIP data were used to present national estimates of crash injury rates because this system focuses on injury-related visits to EDs, where most crash injuries are initially treated. Second, work-related crashes might not have been identified consistently and could be undercounted. Third, the lifetime medical cost estimates presented in this report did not include medical costs among patients that left against medical advice or were kept for observation without hospital admission; however, only 1% of the NEISS-AIP sample fell into this category. Finally, the cost estimates represent less than the full identifiable economic burden because this report does not include costs such as property damage.

This analysis suggests that states, employers, and individuals can avert substantial medical costs by adopting safety practices and policies shown to protect motor vehicle occupants. Primary seatbelt laws, child passenger restraint laws, ignition interlocks to prevent alcohol impaired driving, publicized sobriety checkpoints, and graduated driver licensing systems for teens all have demonstrated effectiveness to reduce crash injuries and fatalities (14–18). These interventions reduce injuries and result in economic savings. For instance, an estimated 54,000 serious injuries could be prevented annually if all occupants wore seatbelts, and 82,000 serious injuries could be prevented if all drivers had a blood alcohol content of <0.08 g/dL (19). The 2009 passage of a primary seat belt law in Minnesota is estimated to have increased seat belt use and averted $45 million in hospital charges, or roughly an estimated $36 million in hospital costs (Healthcare Cost and Utilization Project, unpublished data, 2010) over a 2-year period (20). The presence of graduated driver licensing laws is associated with reduced injuries and reduced cost for private and public payers (14). A $30 booster seat is estimated to save an average of $245 in medical costs over 4 years of use (21). Finally, publicized sobriety checkpoint programs show benefit-cost ratios ranging from 2:1 to 57:1 (15). To date no state has implemented all of these safety measures in accordance with evidence and expert recommendation (22).

Nonfatal crash injuries occur frequently, resulting in substantial costs to individuals, families, employers, and society. In recognition of the impact of these injuries, the Moving Ahead for Progress in the 21st Century Act (23) requires states to monitor serious crash injuries, in addition to fatalities, to receive full highway funding. Comprehensive data on nonfatal crash injuries will improve the ability of government, employers, and health and traffic safety organizations to understand and prevent motor vehicle crash injuries. Ultimately, full implementation of effective interventions will reduce the health and cost burden from crash injuries.

**Key Points**

- In 2012, an estimated 2,519,471 emergency department visits resulting from nonfatal crash injuries occurred in the United States, with 7.5% of these visits resulting in hospitalization, accounting for an estimated 1,057,465 hospitalization days in 2012.
- The estimated total lifetime medical cost of nonfatal crash injuries was $18.4 billion (in 2012 dollars), consisting of $7.7 billion among patients treated and released from the emergency department and $10.7 billion among hospitalized patients.
- Teens and young adults aged 15–29 years account for 21% of the population but accounted for 38% of the costs for patients treated and released for crash injuries.
- Primary seatbelt laws, child passenger restraint laws, ignition interlocks to prevent alcohol impaired driving, publicized sobriety checkpoints, and graduated driver licensing systems for teens all have shown effectiveness to reduce crash injuries and fatalities. To date, no state has implemented all of these safety measures in accordance with evidence and expert recommendation.
- Additional information is available at http://www.cdc.gov/vitalsigns.

## Figures and Tables

**TABLE 1:** Number and rate* of emergency department visits for nonfatal crash injuries among motor vehicle occupants, by age group, sex, and disposition — National Electronic Injury Surveillance System, United States, 2012

Age group and sex	Total†			Treated and released			Hospitalized		
		
	No. of visits§¶	No. per 100,000	(95% CI)	No. of visits§¶	No. per 100,000	(95% CI)	No. of visits§¶	No. per 100,000	(95% CI)
**0–14 yrs**									
Total	171,954	281.2**	(218.1–344.4)	160,810	263.0	(203.5–322.5)	8,315	13.6	(7.2–20.0)
Female	94,152	314.9	(244.0–385.8)	88,790	296.9	(228.8–365.1)	4,241	14.2	(8.0–20.4)
Male	77,802	249.0	(191.8–306.3)	72,020	230.5	(178.0–283.0)	4,074	13.0	(5.9–20.2)
**15–29 yrs**									
Total	949,524	1,447.6**	(1,164.7–1,741.6)	877,366	1,342.7	(1,074.5–1,611.0)	60,737	93.0	(49.5–136.4)
Female	535,478	1,669.0	(1,330.7–2,017.6)	504,770	1,578.1	(1,250.4–1,905.9)	25,042	78.6	(41.2–115.4)
Male	414,022	1,235.5	(999.4–1,482.9)	372,572	1,116.9	(900.0–1,333.9)	35,696	107.0	(56.9–157.1)
**15–17 yrs**									
Total	124,977	993.1	(772.1–1,214.2)	114,047	906.3	(703.9–1,108.7)	9,408	74.8	(36.0–113.5)
Female	72,566	812.9	(917.5–1,447.4)	67,818	1,105.1	(856.0–1,354.2)	3,740	60.9	(27.0–94.8)
Male	52,411	812.9	(624.5–1,001.4)	46,229	717.0	(549.7–884.4)	5,668	87.9	(42.6–133.2)
**18–20 yrs**									
Total	239,563	1,798.0	(1,395.4–2,201.5)	219,644	1,648.9	(1,287.5–2,010.4)	17,106	128.4	(62.6–194.3)
Female	134,161	2,074.0	(1,608.6–2,538.8)	125,761	1,943.8	(1,511.6–2,376.1)	7,055	109.0	(47.3–170.8)
Male	105,402	1,539.0	(1,181.4–1,895.6)	93,883	1,370.4	(1,060.3–1,680.5)	10,051	146.7	(73.8–219.7)
**21–24 yrs**									
Total	292,060	1,619.0	(1,288.0–1,950.0)	269,885	1,496.1	(1,186.3–1,805.9)	18,074	100.2	(53.2–147.2)
Female	166,130	1,882.5	(1,453.3–2,311.7)	156,774	1,776.5	(1,368.5–2,184.4)	7,690	87.1	(43.5–130.8)
Male	125,905	1,366.4	(1,114.8–1,618.0)	113,087	1,227.3	(997.8–1,456.8)	10,384	112.7	(60.2–165.2)
**25–29 yrs**									
Total	292,925	1,368.9	(1,096.6–1,641.3)	273,790	1,279.5	(1,016.9–1,542.1)	16,150	75.5	(40.3–110.6)
Female	162,620	1,540.9	(1,231.6–1,850.2)	154,417	1,463.2	(1,160.0–1,766.4)	6,558	62.1	(34.9–89.4)
Male	130,304	1,201.5	(955.7–1,447.3)	119,373	1,100.7	(869.0–1,332.5)	9,592	88.5	(43.6–133.3)
**30–39 yrs**									
Total	434,428	1,075.3	(883.3–1,267.3)	407,260	1,008.1	(817.7–1,198.5)	23,556	58.3	(33.9–82.7)
Female	242,240	1,199.8	(986.4–1,413.1)	229,945	1,138.9	(926.2–1,351.5)	10,169	50.4	(30.5–70.2)
Male	192,188	951.0	(766.5–1,135.6)	177,315	877.4	(696.1–1,058.8)	13,387	66.2	(36.1–96.4)
**40–49 yrs**									
Total	368,556	862.8	(683.9–1,041.6)	341,140	798.6	(621.0–976.2)	23,608	55.3	(31.1–79.5)
Female	202,933	942.5	(748.1–1,136.8)	192,064	892.0	(697.5–1,086.5)	9,628	44.7	(22.2–67.2)
Male	165,624	781.8	(612.4–951.2)	149,076	703.7	(538.3–869.0)	13,980	66.0	(38.9–93.0)
**50–59 yrs**									
Total	304,965	703.0	(576.0–831.0)	275,930	636.5	(514.1–758.9)	25,548	58.9	(37.0–80.9)
Female	169,333	763.0	(619.9–905.4)	156,938	706.8	(569.1–844.6)	10,839	48.8	(30.4–67.2)
Male	135,631	641.0	(522.1–760.5)	118,992	562.6	(449.2–676.1)	14,710	69.6	(42.5–96.6)
**60–69 yrs**									
Total	167,330	526.3	(414.1–638.6)	146,687	461.4	(364.4–558.4)	18,813	59.2	(35.2–83.2)
Female	95,216	571.9	(448.6–695.2)	85,188	511.6	(398.8–624.5)	9,170	55.1	(34.7–75.4)
Male	72,114	476.3	(372.6–580.0)	61,499	406.2	(324.3–488.0)	9,644	63.7	(34.0–93.4)
**70–79 yrs**									
Total	78,389	448.0	(351.0–545.0)	63,970	365.6	(292.5–438.7)	13,515	77.2	(46.4–108.0)
Female	46,286	481.6	(366.2–597.1)	37,865	394.0	(305.7–482.3)	7,900	82.2	(50.5–113.9)
Male	32,103	407.0	(321.4–492.7)	26,105	331.0	(266.9–395.1)	5,615	71.2	(37.8–104.6)
**≥80 yrs**									
Total	44,223	378.9	(267.7–490.1)	29,035	248.8	(183.1–314.5)	14,648	125.5	(68.0–183.0)
Female	26,509	360.7	(261.2–460.2)	17,562	239.0	(174.1–303.8)	8,783	119.5	(66.2–172.8)
Male	17,714	410.0	(268.8–551.1)	11,473	265.5	(186.7–344.4)	5,866	135.8	(65.3–206.2)
**All ages** ††									
Total	2,519,471	806.3	(757.7–854.9)	2,302,207	738.5	(692.2–784.7)	188,833	58.8	(51.5–66.1)
Female	1,412,180	901.5	(844.9–958.2)	1,313,130	841.2	(786.6–895.9)	85,794	51.9	(45.3–58.5)
Male	1,107,268	712.7	(669.3–756.2)	989,053	637.1	(596.6–677.6)	103,039	65.9	(57.3–74.5)

**Abbreviation:** CI = confidence interval.

* Per 100,000 population.

† Total estimates include patients with disposition coded as “observed,” “left against medical advice,” or “unknown.”

§ National estimates based on weighted data from the National Electronic Injury Surveillance System – All Injury Program.

¶ Totals include visits with unknown age and/or unknown sex. Estimates might not add up to total because of rounding.

** Rate is significantly different compared with other age groups within the same disposition category.

†† Estimates for all ages are age-adjusted.

**TABLE 2:** Average and total costs* of emergency department visits for nonfatal crash injuries among motor vehicle occupants, by age group, sex, and disposition — National Electronic Injury Surveillance System, United States, 2012

	Treated and released			Hospitalized		
		
Age group and sex	No. of visits†§	Average cost ($)	Total cost ($)	No. of visits†§	Average cost ($)	Total cost ($)
**0–14 yrs**						
Total	160,810	3,370	541,913,000	8,315	63,738	529,983,000
Female	88,790	3,472	308,311,000	4,241	61,929	262,641,000
Male	72,020	3,244	233,602,000	4,074	65,622	267,342,000
**15–29 yrs**						
Total	877,366	3,386	2,971,125,000	60,737	58,220	3,536,130,000
Female	504,770	3,278	1,654,612,000	25,042	48,815	1,222,416,000
Male	372,572	3,534	1,316,513,000	35,696	64,817	2,313,714,000
**30–39 yrs**						
Total	407,260	3,239	1,319,055,000	23,556	56,703	1,335,693,000
Female	229,945	3,020	694,399,000	10,169	51,096	519,593,000
Male	177,315	3,523	624,656,000	13,387	60,962	816,100,000
**40–49 yrs**						
Total	341,140	3,311	1,129,637,000	23,608	53,405	1,260,796,000
Female	192,064	3,106	596,617,000	9,628	53,063	510,886,000
Male	149,076	3,575	533,020,000	13,980	53,642	749,910,000
**50–59 yrs**						
Total	275,930	3,315	914,703,000	25,548	53,638	1,370,351,000
Female	156,938	3,178	498,816,000	10,839	51,806	561,529,000
Male	118,992	3,495	415,887,000	14,710	54,984	808,822,000
**60–69 yrs**						
Total	146,687	3,507	514,419,000	18,813	55,378	1,041,821,000
Female	85,188	3,593	306,085,000	9,170	48,115	441,218,000
Male	61,499	3,388	208,334,000	9,644	62,277	600,603,000
**70–79 yrs**						
Total	63,970	3,783	241,970,000	13,515	59,011	797,531,000
Female	37,865	3,866	146,392,000	7,900	53,432	422,114,000
Male	26,105	3,661	95,578,000	5,615	66,860	375,417,000
**≥80 yrs**						
Total	29,035	3,679	106,829,000	14,648	56,103	821,795,000
Female	17,562	3,754	65,924,000	8,783	52,191	458,391,000
Male	11,473	3,565	40,905,000	5,866	61,951	363,404,000
**All ages**						
Total	2,302,207	3,362	7,739,677,000	188,833	56,674	10,701,947,000
Female	1,313,130	3,253	4,271,182,000	85,794	51,279	4,399,393,000
Male	989,053	3,507	3,468,495,000	103,039	61,167	6,302,554,000

* Costs are in 2012 U.S. dollars.

† National estimates based on weighted data from the National Electronic Injury Surveillance System – All Injury Program.

§ Totals include visits with unknown age and/or unknown sex. Estimates might not add up to total because of rounding.
